# Single center experience with laparoscopic adrenalectomy on a large clinical series

**DOI:** 10.1186/s12893-017-0333-8

**Published:** 2018-01-11

**Authors:** Giovanni Conzo, Claudio Gambardella, Giancarlo Candela, Alessandro Sanguinetti, Andrea Polistena, Guglielmo Clarizia, Renato Patrone, Francesco Di Capua, Chiara Offi, Mario Musella, Sergio Iorio, Giseppe Bellastella, Daniela Pasquali, Annamaria De Bellis, Antonio Sinisi, Nicola Avenia

**Affiliations:** 1 Division of General and Oncologic Surgery, Department of Cardiothoracic and Respiratory Sciences, University of Campania “Luigi Vanvitelli”, Via Pansini 5, 80131 Naples, Italy; 2 Endocrine Surgery Unit, University of Perugia, Piazza dell’Università, 06123 Perugia, Italy; 3 Department of Medical, Surgical, Neurological, Metabolic and Aging Sciences, University of Campania “Luigi Vanvitelli”, Caserta, Italy; 4 Advanced Biomedical Sciences Department, Federico II University, Napoli, Italy

**Keywords:** Laparoscopic adrenalectomy, Pheochromocytoma, Minimally invasive adrenalectomy, Cushing syndrome, Incidentaloma

## Abstract

**Background:**

Laparoscopic adrenalectomy is considered the gold standard technique for the treatment of benign small and medium size adrenal masses (<6 cm), due to low morbidity rate, short hospitalization and patient rapid recovery. The aim of our study is to analyse the feasibility and efficiency of this surgical approach in a broad spectrum of adrenal gland pathologies.

**Methods:**

Pre-operative, intra-operative and post-operative data from 126 patients undergone laparoscopic adrenalectomy between January 2003 and December 2015 were retrospectively collected and reviewed. Diagnosis was obtained on the basis of clinical examination, laboratory values and imaging techniques. Doxazosin was preoperatively administered in case of pheochromocytoma while spironolactone and potassium were employed to treat Conn’s disease. Laparoscopic adrenalectomies were all performed by the same surgeon (CG). First 30 procedures were considered as learning curve adrenalectomies.

**Results:**

One hundred twenty-six patients were included in the study. Functioning tumors were diagnosed in 84 patients, 27 patients were affected by pheochromocytomas, 29 by Conn’s disease, 28 by Cushing disease. Surgery mean operative time was 137.33 min (range 100–180) during the learning curve adrenalectomies and 96.5 min (range 75–110) in subsequent procedures. Mean blood loss was respectively 160.2 ml (range 60–280) and 90.5 ml (range 50–200) in the first 30 procedures and the subsequent ones. Only one conversion to open surgery occurred. No post-operative major complications were observed, while minor complications occurred in 8 patients (0,79%). In 83 out of 84 functioning neoplasms, laparoscopic adrenalectomy was effective in normalization of endocrine profile.

**Conclusions:**

Laparoscopic adrenalectomy is a safe and feasible procedure, even for functioning masses and pheochromocytomas. A multidisciplinary team including endocrinologists, endocrine surgeons and anaesthesiologists, is recommended in the management of adrenal pathology, and adrenal surgery should be performed in referral high volume centers. A thirty-procedures learning curve is recommended to improve surgical outcomes.

## Background

Nowadays different surgical approaches are available to treat adrenal masses, any of them showing strengths and weaknesses, and the choice of any approach depends on different parameters (such as tumor size, malignancy, patient’s conditions, surgical experience).

Large malignant adrenocortical tumors, due to haemodynamic instability and the risk of tumor cell dissemination, are worldwide considered as indications to “open surgery”, that is unavoidably associated to greater postoperative pain and longer hospitalization causing higher morbidity and mortality rate [1]. Laparoscopic adrenalectomy (LA) by transabdominal lateral approach is considered a gold standard technique to treat a broad spectrum of functioning and non-functioning adrenal diseases, with described cases of resection of masses up to 12–15 cm [2, 3]. Also robotic adrenalectomy and posterior retroperitoneal adrenalectomy (PRA) [4] represent excellent minimal invasive options, especially for benign and medium sized (<6 cm) tumors. Low morbidity rate, short hospitalization, improved cosmesis and a rapid recovery in addition to increasing patients satisfaction and comfort are the main well known advantages [5].

Currently indication to LA for lesions >6 cm is still a matter of debate and experienced endocrine surgeons are divided between supporters [6–8] and detractors [9].

The Authors retrospectively reviewed data about 126 patients, undergone LA in endocrine academic referral center, in order to evaluate safety and outcomes of laparoscopic procedures in the treatment of adrenal pathologies. Literature review was performed and collected data compared to those described in the main published series.

## Methods

### Study design

All clinical cases of patients undergone LA for adrenal neoplasm between January 2003 and December 2015 were retrospectively reviewed. Diagnosis was obtained on the basis of clinical examination, laboratory values and imaging techniques (ultrasonography, computed tomography and Magnetic Resonance Imaging) [10]. ASA score ≤ III, age less than 80 years old, adrenal benign functioning tumors up to 8 cm in diameter, non-functioning tumors ≤12 cm, and adrenal metastases <6 cm were the main surgical inclusion criteria. Patients with suspected primary malignant adrenal neoplasm, on the basis of imaging examinations (local invasion or metastases), were addressed to open surgery and not enrolled in the study [11]. Patients with definitive pathology of primary malignant tumor were excluded from the study. Patient’s evaluation included pre-operative, intra-operative and post-operative data. Surgical procedures were all performed by the same surgeon (CG), experienced in endocrine and laparoscopic surgery, assisted by a collaborative team skilled in minimal invasive surgery. The first 30 patients were considered as part of the learning curve [12–14].

### Pre-operative management

Before surgery, all patients underwent laboratory tests, chest X-ray, ECG examination and cardiological counseling. A scheduled pharmacological treatment was administrated in case of pheocromocytomas (PCC) and Conn syndrome. All patients were administered antithrombotic prophylaxis (heparin sodium 4000 U.I. s.c.). Patients diagnosed with PCC underwent an alpha-1 blocker – doxazosin 2 mg (3 cps per day), until blood pressure (BP), heart rate (HR) and ECG stabilization were achieved, (BP < 160/90 mmHg and HR < 100 beats min for at least 24 h before surgery, ECG ST normalization for at least 1 week before surgery). In case of cardiological stabilization failure, a beta-blocker (Atenolol 50 mg one cp per day) was administered as well [15]. In each PCC case plasma volume was preoperatively expanded using crystalloid solutions.

Patients affected by Conn’s disease with low potassium serum level, preoperatively were administered with spironolactone 50 mg (1 to 6 cps per day) and Potassium Aspartate 3 mEq/ml i.v. (one vial per day) in order to reach normal range blood Potassium levels (n.r. 3,5–5 mmol/l).

### Intra-operative management

All patients received general anesthesia with orotracheal intubation and antibiotic prophylaxis (cefazoline 2 g i.v.) was performed. Anesthesia chart and pathology report of each patient were collected. Systolic blood pressure (SBP) levels ≥180 mmHg were considered hypertensive crises, while SBP levels <90 mmHg were considered hypotensive ones.

LA were performed using a standard transperitoneal lateral laparoscopic approach. The patients were placed in the left position (30° angle) for right LA, while they were placed in right position (90° angle) for left LA. Right LA was approached using 4 trocars, while left LA was approached with 3 trocars. Pneumoperitoneum was maintained at 12–14 mmHg by insufflation of carbon dioxide (CO2). Dissection was realized using Harmonic scalpel™ (Ethicon Endo Surgery INC - Johnson & Johnson, NJ, USA) or LigaSure ™ vessel sealing system (Tyco, Boulder CO, USA). The first step consisted of vascular control of the main adrenal vein by clips. Whenever necessary, the lateral and posterior connections of the right hepatic lobe were incised and the liver was superiorly and medially retracted, paying particular attention in Cushing’s patients, in whom the liver may be very fragile. For right adenomas larger than 6 cm, adrenal gland was mobilized on both the upper and medial sides, in order to have a safer access to the inferior cava vein and the ipsilateral renal vein. In left LA, a wide left colon mobilization was routinely carried out. The surgical specimens were extracted in retrieval bags through a mini-laparotomy of the trocars. A 20 Fr drainage was routinely placed.

### Post operative management

After surgery all patients were administered with fluids (1000 ml of saline solution NaCl 0.9% + 1000 ml of polysaline solution +500 ml of glucose solution 5% i.v.) and antithrombotic therapy (heparin sodium 4000 U.I. s.c.) until discharge. Postoperative hypotensive crises were treated by hydrocortisone and Crystalloids infusions i.v. Early mobilization and feeding were recommended on the first postoperative day. Drainage was removed on the first or second post operative day. Major and minor complications were recorded in patients’clinical folders.

Patients’ follow up consisted of clinical evaluation and blood examination at the day 7–15-30 after discharge, followed by half-yearly controls.

### Statistics

Data were expressed as mean, unless otherwise specified. Statistical analysis was performed with SPSS version 11.5 (SPSS©, Chicago, IL, USA). Significance was assigned with a *p* value <0.05.

## Results

From January 2003 until December 2015, 126 patients (38 males, 88 females) were enrolled in the study. The mean age was of 51.7 years old (range 22–76). Patients comorbidities are reported in Table 1. A Right neoplasm was present in 59% of the cases, while one patient presented bilateral masses. Functioning tumors were diagnosed in 84 patients (67%), 27 patients were affected by PCC, 29 by Conn’s disease, 28 by Cushing. In 38 cases diagnosis was incidental, while one patient was diagnosed as myelolipoma. Three patients presented adrenal metastasis after being treated for breast and renal cancers respectively (Table 2). Mean size of adrenal neoplasm was 5.75 cm (range 1.1 to 12 cm). According to anaesthesiology counselling, 51 patients had ASA score I, 56 had ASA II score, 19 patients were classified ASA III. PCC patients well tolerated pre-operative therapy with doxazosine, resulting in normalization of BP, HR and ECG test after a mean time of 8.6 days (range 4 to 15 days). One patient needed beta-blocker supplementary administration in order to reach pre-operative goals. Five patients affected by Conn’s disease needed supplementary Potassium administration in order to reach normal range of potassium levels.

**Table 1:** Demographics

		Percent
Mean age (years)	51.7	
Male patients (n)	38	30.2
Female patients (n)	88	69.8
Cardiovascular disease (n)	35	27.8
Pulmonary disease (n)	20	25.2
ASA score 1–2 (n)	107	84.9
ASA score 3 (n)	19	15.1

**Table 2:** Adrenal neoplasms’ characteristics

		Percent
Right site (n)	74	59
Left site (n)	51	40.2
Bilateral site (n)	1	0.79
Mean size (cm)	5.75 cm	
PCC (n)	27	21.43
Conn’s (n)	29	23
Cushing (n)	28	22.22
Incidental (n)	38	30.16
Metastasis (n)	3	2.4
Myelolipoma (n)	1	0.79

Regarding surgery, mean operative time was 137.33 min (range 100–180) during the learning curve and 96.5 min (range 75–110) in subsequent procedures. Mean blood loss was 160.2 ml (range 60–280) in first 30 procedures and it lowered to 90.5 ml (range 50–200) in subsequent procedures (Table 3). No patient required intra or postoperative blood transfusion. Both mean operative time and mean blood loss resulted a statistically significant difference between learning curve and total operations (*p* < 0.05). Among intra-operative complications, it was reported one case of inferior vein cava injury, sutured and treated by Floseal® Hemostatic Matrix (Baxter Zurich Switzerland) and oxidized cellulose (Tabotamp Fibrillar Johnson & Johnson, NJ, US), and one case of diaphragm lesion due to pleural cavity opening, both successfully treated without necessity of conversion to open surgery. No cases of splenic or liver injuries occurred in our series. It has been reported only one case of conversion to open surgery (0.79%), due to suspected infiltration of the renal vessels by a left PCC, not confirmed at definitive pathology, which reported a desmoplastic reaction.

**Table 3:** Peri-operative data

	Learning Curve (30)^b^	Subsequent Procedures (96)
Mean operative time (min)	137.33 (range 100–180)	96.5(range 75–110)*
Mean Intraoperative blood loss (ml)	160.2 (range 60–280)	90.5 (range 50–200)*(*p* < 0,01)
Hypertensive crises (SBP > 180 mmHg)	4/30 (13.3%)	12/96 (12.5%)
Hypotensive crises (SBP < 90 mmHg)	0/30 (0%)	4/96 (4.1%)
Conversion to open procedure (n)	1/30 (3.3%)	0/96 (0%)
30-day morbidity^a^ (n)	3/30 (10%)	5/96 (5.2%)*(*p* = 0,025)
Mean Hospital stay (days)	4.5 (3–8 days)	3.4 (range 2–10 days)

Legend: (n) = number; (cm, ml, mmHg) = measurements; (min, days) = time

*statistically significant difference (*p* < 0,05)

^a^abdominal wall hematoma, pneumonia, port site hernia, intra-abdominal collection

^b^30 procedures were considered learning curve, basing on literature [12–14]

Intraoperative hypertensive crises were reported in 16 cases (93% in PCC patients, 7% in Cushing patient), 3 at the induction, 13 during adenoma manipulation (four cases during the learning curve), while hypotensive crises were reported in 4 cases (one severe hypotension), but they were promptly and successfully treated without leading to post-operative injuries. Cardiac enzymes increasing in absence of myocardial infarction clinical manifestations was observed in one female PCC patient.

At definitive pathology, no occurrence of malignant primary tumor was observed (0/126).

No postoperative mayor complication occurred. 30-day morbidity rate was 6.3% (8/126 patients) with a statistically significant difference between learning curve and subsequent operations, and consisted of three cases of abdominal wall hematoma (two cases during the learning curve), two cases of port site hernia (one case during the learning curve), one case of pneumonia and two intra-abdominal collection spontaneously resolved (Table 3).

Mean hospitalization was 3.4 days (2–10 days). Mean follow up was 47.78 months (6–160 months). Surgery determined an hormonal serum levels normalization in 83 out of 84 diagnosed with functional adenoma (98.8%); only one patient affected by PCC disease showed a persistent postoperative hypertension and elevation of metanephrine levels, due to retrocaval adrenal tissue, requiring reoperation with posterior approach.

A male patient affected by Conn’s disease was diagnosed of a methacronous controlateral tumor about 8 years after a right LA, and was submitted to a sparing ultrasound-assisted left LA. No complication and/or disease recurrence were observed at 54.43 month follow-up (6–120 months),. Follow up in three female patients, affected by breast and renal cancers, stated that they are alive respectively 1, 2 and 4 years following LAs.

## Discussion

Since the first successfully performed LA by Gagner in 1991 [16], the transperitoneal approach has become the most common therapeutic strategy for adrenal neoplasm [17]. Thanks to the minimal invasive approach, LA allowed a decreased postoperative pain, a reduced ileus, a shorter hospitalization, an earlier return to work and a better cosmetic result, guarantying a lower morbidity (5 to 20%) and mortality rates (below 0,5%) [18–23]. OA, associated to higher mortality (2–4%) and morbidity rates (bleeding, pulmonary and cardiac issues, pulmonary thromboembolism, wound infections), is worldwide reserved only for large tumors (diameter > 6 cm) and primary malignancies, based on the radicality of resection, minor tumor local recurrence, and major survival [24–26]. Suspected primary malignant adrenal tumors should be considered as contraindication to minimal invasive approach, for the poor oncological outcome and the high risk of peritoneal dissemination of primary adrenal cancer. Nevertheless, the best surgical approach is still a matter of debate [27–30]. Several authors underline how family history, virilising features, mixed hormonal secretion, rapid enhancement and rapid washout on MRI contrast imaging are considered predictor factors of malignancy, nevertheless only local invasion or metastases are clear signs of adrenal cancer [11]. Basing on these surgical principles, in our experience, thanks to a careful and efficient anamnesis, together with laboratory and imaging evaluation, no specimen was found malignant at definitive pathology.

According to literature data [20, 31–33], LA is indicated in patients affected by PCC up to 8 cm, incidentalomas up to 6 cm and metastases <6 cm. Thanks to the use of ultrasound or radiofrequency dissection, we also demonstrated that minimal invasive approach can be safely performed by experienced surgeons also in the treatment of benign tumors larger than 6 cm and in case of metastases, extending the previous surgical indications [5, 6, 34, 35]. Regarding devices efficacy, in our series we did not observe significant differences between Harmonic scalpel™ (Ethicon Endo Surgery INC - Johnson & Johnson, NJ, USA) and LigaSure ™ vessel sealing system (Tyco, Boulder CO, USA), nevertheless we are more experienced with LigaSure ™. We retain instead, as mandatory parameter, that LA should be assessed by experienced laparoscopic surgeons after a learning curve procedures [18, 36]. Basing on literature data, we assumed the first 30 surgical procedures as learning curve [12–14]. In our series, we observed a significant reduction of mean operative time and intra-operative blood loss comparing the first 30 procedures to the subsequent ones. We referred these improvements to a gained knowledge of anatomical connections, leading to a safer manipulation of the inferior vein cava, a prompter identification of the renal vein in order to identify the medial adrenal vein (in left LA) and a more efficient mobilization and retraction of the liver (in right LA). Also post-operative complication rate resulted significantly reduced, despite only subclinical sequelae occurred even during the learning curve procedures.

Surgical approach was effective, safe and well tolerated, with a negligible morbidity rate. We reported only one case of conversion to open surgery (0.79%), due to suspected infiltration of the renal vessels by a left PCC, not confirmed at definitive pathology, which reported a desmoplastic reaction. In our series, a conversion to open surgery, following intraoperative complications was not necessary, and a “conservative” management of inferior vena cava and diaphragm injuries were easily performed, without excessive risks for the patients. However, in case of intraoperative risky complications a rapid conversion is mandatory to avoid life threatening events [18].

A preoperative selective adrenergic blockade with doxazosin, routinely performed, did not prevent intraoperative hypertensive episodes (16 cases), occurring principally during tumor manipulation (13 out of 16 cases), and promptly treated by anaesthesiologists. Major intraoperative cardiovascular complications - cerebral vascular accident, pulmonary edema, myocardial infarction or ischemia, cardiac arrhythmias and multiorgan failure - were not observed, although it is reported that laparoscopic treatment for metastases or PCCs present high risk for hemodynamic disorders [37]. Large indeterminate adrenal neoplasm, carcinoma, a tumor size >6 cm and concomitant surgical procedures are considered the main risk factors predicting 30-day morbidity [32]. In Cushing’s diseases, a higher post-operative infectious complication rate was not observed.

The presented retrospective study has several limitations. All operations were performed by a single team, composed by experienced endocrine and laparoscopic surgeons, in a tertiary referral center. The study design was retrospective and a comparison with an “open surgery” series was not carried out. Nevertheless it is possible to drawn some conclusions.

## Conclusion

A multidisciplinary routinely management - endocrinologists, endocrine surgeons and anaesthesiologists - is recommended in referral high volume units for the treatment of adrenal pathology. An accurate pre-operative examination is mandatory due to select eligible patients to LA. LA is safe and feasible also for benign lesions up to 12 cm. A skilled operative team, composed by endocrine surgeons experienced in LA after adequate learning curve, is requested. Preoperative alfa-blockade does not prevent PCC hypertensive crises but, facilitating their pharmacological control, must be recommended.

## Abbreviations

BP: Blood pressure
HR: Heart rate
LA: Laparoscopic adrenalectomy
OA: Open adrenalectomy
PCC: Pheochromocytoma
PRA: Posterior retroperitoneoscopic adrenalectomy
SBP: Systolic blood pressure
